# Stratification of the severity of critically ill patients with classification trees

**DOI:** 10.1186/1471-2288-9-83

**Published:** 2009-12-09

**Authors:** Javier Trujillano, Mariona Badia, Luis Serviá, Jaume March, Angel Rodriguez-Pozo

**Affiliations:** 1 Intensive Care Unit, Hospital Universitario Arnau de Vilanova, IRBLLEIDA, (Avda Rovira Roure 80), Lleida (25198), Spain; 2 Departamento de Ciencias Médicas Básicas, Universidad de Lleida, (Avda Rovira Roure 44), Lleida (25006), Spain; 3 Departamento de Cirugía, Universidad de Lleida, (Avda Rovira Roure 80), Lleida (25198), Spain

## Abstract

**Background:**

Development of three classification trees (CT) based on the CART (*Classification and Regression Trees*), CHAID (*Chi-Square Automatic Interaction Detection*) and C4.5 methodologies for the calculation of probability of hospital mortality; the comparison of the results with the APACHE II, SAPS II and MPM II-24 scores, and with a model based on multiple logistic regression (LR).

**Methods:**

Retrospective study of 2864 patients. Random partition (70:30) into a Development Set (DS) n = 1808 and Validation Set (VS) n = 808. Their properties of discrimination are compared with the ROC curve (AUC CI 95%), Percent of correct classification (PCC CI 95%); and the calibration with the Calibration Curve and the Standardized Mortality Ratio (SMR CI 95%).

**Results:**

CTs are produced with a different selection of variables and decision rules: CART (5 variables and 8 decision rules), CHAID (7 variables and 15 rules) and C4.5 (6 variables and 10 rules). The common variables were: inotropic therapy, Glasgow, age, (A-a)O2 gradient and antecedent of chronic illness. In VS: all the models achieved acceptable discrimination with AUC above 0.7. CT: CART (0.75(0.71-0.81)), CHAID (0.76(0.72-0.79)) and C4.5 (0.76(0.73-0.80)). PCC: CART (72(69-75)), CHAID (72(69-75)) and C4.5 (76(73-79)). Calibration (SMR) better in the CT: CART (1.04(0.95-1.31)), CHAID (1.06(0.97-1.15) and C4.5 (1.08(0.98-1.16)).

**Conclusion:**

With different methodologies of CTs, trees are generated with different selection of variables and decision rules. The CTs are easy to interpret, and they stratify the risk of hospital mortality. The CTs should be taken into account for the classification of the prognosis of critically ill patients.

## Background

Stratifying the patients into risk groups, according to their severity, is essential for the comparison of treatments and the establishment of differences between different units or hospital centres. As a result, working in an intensive care unit (ICU) necessitates making prognoses for patients within the first 24 hours of their admission. Establishing a prognosis consists of assigning a probability of death by using variables commonly used for the diagnosis and treatment of critically ill patients [1].

Severity scores are classic tools used in establishing this probability. The most commonly used scores are the APACHE II (*Acute Physiology and Chronic Health Evaluation II*), the SAPS II (*Simplified Acute Physiology Score II*) and the MPM II-24 (*Mortality Probability Models II-24*) scores [2-4].

Other systems of severity classification based on different mathematical strategies have also been used [5].

In the last decade, classification trees (CT), which were developed more than 20 years ago, have acquired greater importance in the immediate interpretation of the decision rules that they generate, and they are readily accepted by professionals in clinical practice [6].

A CT is a graphic representation of a series of decision rules. Beginning with a root node that includes all cases, the tree branches are divided into different child nodes that contain a subgroup of cases. The criterion for branching (or partitioning) is selected after examining all possible values of all available predictive variables. In the terminal nodes (the "leaves" of the tree), a grouping of cases is obtained, such that the cases are as homogeneous as possible with respect to the value of the dependent variable [7].

The different CT types are distinguished by the manner of node partitioning. In the specific case of CARTs (*Classification And Regression Trees*), possibly the most widely used CT in medicine, an impurity function (the so-called *Gini *index) is calculated, and for each division of the tree, the variable and its cut-off value are defined such that the decrease in the impurity function is the greatest [8]. There are many types of CTs (or improved versions) such as CHAID (*Chi-square Automatic Interaction Detection*) and C4.5 (developed from the so-called *Concept Learning Systems*). Table 1 illustrates, in a schematic fashion, the particularities of these CTs. A CT has a growth phase, a pruning phase (removal of branches that do not provide general information to the system) and a selection of the optimal tree [8].

**Table 1:** Characteristics of the classification tree methods

	CART	CHAID	C4.5
**Description**	Classification and Regression Tree	Chi-Square Automatic Interaction Detection	Concept Learning Systems Version 4.5

**Developer**	Breiman et al. (1984)	Kass (1980)	Quinlan (1993)

**Primary Use**	Many disciplines with little data	Applied statisticians	Data miners

**Splitting Method**	Gini reduction or twoing	Chi-square tests F test	Gain ratio

**Branch Limitations**	Best binary split	Number of values of the input	Best binary split

**Pruning**	Cross-validation	Uses p-values	Misclassification rates

**Programs**	WEKA DTREG Answer-Tree (SPSS)	Answer-Tree (SPSS)	WEKA

The aim of the present study was to develop (with a population of critically ill patients) three classification trees (based on CART, CHAID and C4.5 methodologies) to calculate the probability of hospital mortality and to compare these trees with each other, with the classic scores (APACHE II, SAPS II and MPM II-24) and with a model based on multiple logistic regression.

## Methods

This is a retrospective study carried out using the database of a mixed ICU (with medical and surgical services) of 14 beds located at the University Hospital Arnau de Vilanova of Lleida. The ethical committee of the hospital was informed that the study was being carried out, and informed consent was not deemed necessary, since all the variables were collected for the diagnosis and treatment of the patients and their anonymity was assured at all times.

### Database

Data collected over ten years (from January 1997 to December 2006) were used. In this study, all patients were over the age of 16 years and remained in the ICU for more than 24 hours. Patient records with incomplete data were not used.

A random partition, in a 70:30 ratio, was made to establish the development and the validation sets, respectively.

Data concerning age, sex, length of stay in the ICU and procedures specific to the ICU were used. The outcome variable of interest was the probability of hospital mortality. The patients were divided according to their diagnostic groups following the *Knaus *classification [9]. Six diagnostic groups were established according to the case mix and level of severity of the ICU, including two trauma categories of **TBI **(traumatic brain injury) and **Multiple trauma (**multiple trauma without brain injury), **Respiratory **(chronic respiratory problems with decompensation), **Neurological **(ischemic or hemorrhagic strokes), **Surgery **(surgical problems not included in other categories) and **Medicine **(medical pathology not included in other categories).

Each patient's medical records and laboratory database files were used to obtain information pertaining to baseline (at ICU admission) demographics, pre-existing comorbidities and scores (APACHE II, SAPS II and MPM II-24). The data were then compiled (manual recording) into single data using a relational database management system (Microsoft Access©).

APACHE II, SAPS II and MPM II-24 scores were determined by the worst value found during the first 24 hours after ICU admission [2-4].

The presence of acute renal failure was defined (according to the model MPM II-24) by levels of serum creatinin above 2 mg/dL [4]. The antecedents of chronic organ insufficiency (defined according to the APACHE II model) were included in the variable COI [2].

### Logistic models and classification trees

Models were created with the development set and were subsequently checked in the validation set.

Working with the development set, first, a univariate analysis was performed for all the variables included in the three scores to select those that predicted survival. Those that were statistically significant predictors were included in the development of the multivariable models.

We used a model of multiple logistic regression (LR) with forward stepwise selection of variables [10].

The computer programs used for creating the CTs are presented in Table 1. The program WEKA (a project of *Waikato *University) is freely accessible and includes a CT module, named J48, that includes CART and C4.5 [11].

*Answer-Tree©*, a module of SPSS (*Statistical Package for the Social Sciences*), includes options for CART and CHAID, and the program DTREG© (version 3.5) is based on a CART-type methodology.

To create the three types of CTs, a cross-validation system with ten partitions was used, and the only common restriction for terminating the growth of the tree was the minimum number of subjects in the terminal nodes (which was fixed at 50 patients).

### Statistical analysis

The variables are presented as the mean (standard deviation), the median (interquartile interval) or as a percentage. For a comparison of the variables, the chi-squared (χ^2^) test was used for categorical variables, and the ANOVA test or non-parametric *Mann-Whitney *test was used for continuous variables, depending on the characteristics of the distribution.

To compare the different models, we measured their precision (discrimination and calibration) with the *Brier score*. The discrimination was measured by calculating the percentage of correctly classified patients (PCC) with a cut-off point with a probability of 0.5 and by the area below the ROC curve (AUC) [12]. For calibration, the *Hosmer-Lemeshow C *test (HL-C) was used [13] by constructing the calibration curve and calculating the standardized mortality ratio (SMR) [14]. These calculations were made both in the development set and in the validation set. We used a correlation matrix (Spearman correlation coefficients) and the *Bland-Altman *test to analyse the individual probabilities generated by the CT models [15].

The statistical analysis was carried out with the program SPSS (version 14.0).

## Results

### Demographic characteristics

Among 2823 patients, 139 were excluded due to incomplete or erroneous data (4.9%), leaving 2684 eligible patients. The development group consisted of 1880 patients (70%) and the validation group consisted of 804 (30%).

The demographic characteristics of the patients are shown in Table 2; there were no major differences between the development and the validation groups. Some characteristics are particular to the ICU, such as the low proportion of scheduled patients (6.5%), the prolonged length of stay (median of 7 days) and the high mortality rate (31.4%).

**Table 2:** Demographic characteristics of patients

	Group (n = 2684)	Development (n = 1880)	Validation (n = 804)	*p-value*^c^
Age (years)^a^	55.0 (19)	55.2 (19)	54.6 (19)	0.485
Sex, male (%)	66.9	66.8	67.3	0.786
Elective (%)	6.5	6.1	7.5	0.184
				
Diagnostic category				0.414
TBI (%)	15.1	15.2	14.9	
Trauma (%)	15.2	15.2	15.3	
Neurological (%)	14.8	14.6	15.3	
Respiratory (%)	19.0	18.1	21.1	
Surgery (%)	18.7	19.4	17.0	
O Medicine (%)	17.2	17.6	16.3	
				
MV (%)	65.9	66.6	64.2	0.216
Inotropic therapy (%)	33.7	33.9	33.3	0.783
Acute renal failure (%)	19.9	19.8	20.3	0.773
Infection (%)	34.6	34.6	34.8	0.900
Coagulopathy (%)	12.2	12.1	12.6	0.724
COI (%)	16.0	16.3	15.4	0.582
HR^a^	107.8 (30)	108.3 (31)	106.5 (30)	0.253
Glasgow^a^	12.9 (4)	12.8 (4)	13.0 (4)	0.507
(A-a)O2 gradient^a^	244.1 (161)	241.7 (160)	249.5 (162)	0.250
				
APACHE II^b^	18 (7-41)	18 (6-37)	16 (6-45)	0.805
SAPS II^b^	15 (6-47)	15 (5-35)	14 (5-47)	0.742
MPM II-24^b^	17 (7-43)	17 (6-37)	15 (6-38)	0.779
				
LOS (days)^b^	7 (3-16)	7 (3-16)	7 (3-15)	0.972
				
MORT (%)	31.4	30.7	32.8	0.308

TBI: Traumatic brain injury; O Medicine: Other Medical; MV: Mechanical ventilation; A. renal failure: Acute renal failure; COI: Chronic organ insufficiency; HR: Heart rate; (A-a)O2 gradient: Alveolar-arterial oxygen gradient; LOS: Length of stay; MORT: Hospital mortality; ^(a)^: Mean (SD); ^(b)^: Median (Interquartile range) p^c^: *D*etermined by χ^2 ^test for percentages, *t *test for comparison of means or *Mann-Whitney *test for comparison of medians.

Table 3 shows the evolution (during the 10 years observed) of hospital mortality, the severity scores and the participation percentage in the development set. There are no significant differences (only the evidence that the number of admissions has kept on increasing).

**Table 3:** Outcome trend over the observation period

	All	1997	1998	1999	2000	2001	2002	2003	2004	2005	2006	*p-value*^b^
**n**	2684	176	191	201	223	279	297	303	319	337	358	-----
**MORT (%)**	31.4	35.4	33.7	39.1	35.0	28.4	32.9	33.0	28.6	27.7	23.3	0.112
**DEV (%)**	70.0	70.8	61.7	69.0	70.9	72.4	68.9	75.4	66.8	70.4	72.6	0.146
**APACHE II^a^**	18 (7-41)	21 (7-41)	19 (8-41)	19 (6-34)	17 (6-36)	14 (5-30)	14 (6-30)	15 (6-35)	16 (7-38)	17 (6-36)	15 (7-34)	0.361
**SAPS II^a^**	15 (6-47)	15 (6-47)	17 (5-41)	12 (4-31)	13 (3-35)	13 (4-27)	13 (4-31)	15 (5-39)	17 (6-37)	17 (6-37)	13 (5-31)	0.415
**MPM II-24^a^**	17 (7-43)	17 (7-43)	16 (6-35)	14 (6-29)	16 (6-35)	14 (6-32)	13 (6-34)	18 (6-39)	18 (6-40)	17 (7-36)	14 (6-34)	0.389

MORT: Hospital mortality; DEV: Development set percentage ^(a)^: Median (Interquartile range) p^b^: *D*etermined by χ^2 ^test for percentages or *Kruskal-Wallis *test for comparison of medians.

### Variable selection: univariate analysis

A total of 24 variables showed significant differences between the survivors and non-survivors (Table 4). The table also shows the scores for which the different variables were included. No significant differences were found for respiratory frequency (APACHE II), serum potassium (APACHE II and SAPS II), hematocrit (APACHE II), leuckocyte count (APACHE II y SAPS II), bilirubin (SAPS II), PaO2 (MPM II-24) or antecedents of cirrhosis and neoplasia (MPM II-24).

**Table 4:** Univariate analyses of characteristics of patients at discharge, by survival status.

Variable	Survivors (n = 1302)	Non-survivors (n = 578)	*p-value*	*SCORE*
Age (years)	51.2 (19)	63.8 (16)	< 0.001	1,2,3
HR (ppm)	104.7 (29)	115.0 (31)	< 0.001	1,2
MAP (mmHg)	82.8 (28)	72.4 (32)	< 0.001	1,2
Inotropic therapy (%)	25.0	52.7	< 0.001	3
Glasgow	13.4 (3)	11.8 (5)	< 0.001	1,2,3
Intracranial mass (%)	3.0	6.3	0.001	3
FiO2	0.49 (0.2)	0.62 (0.2)	< 0.001	1,2
(A-a)O2 gradient (mmHg)	212.3 (143)	304.5 (176)	< 0.001	1,2
MV (%)	53.6	79.9	< 0.001	3
CO3H (mEq/L)	23.4 (5)	22.1 (6)	< 0.001	1,2
pH	7.36 (0.1)	7.34 (0.1)	< 0.001	1,2
Urine output (cc/24 h)	2124 (1058)	1778 (1398)	< 0.001	2
Urea (mg/dL)	50.8 (41)	76.5 (53)	< 0.001	2
Creatinin (mg/dL)	1.34 (1.3)	1.81 (1.4)	< 0.001	1,3
Sodium (mEq/L)	139.5 (5)	140.5 (7)	0.015	1,2
Acute renal failure (%)	14.1	31.9	< 0.001	3
Urine output < 150 cc/8 h (%)	3.3	17.3	< 0.001	3
Temperature (°C)	38.2 (13)	38.3 (14)	0.036	1,2
Infection (%)	28.8	46.9	< 0.001	3
Coagulopathy (%)	9.7	17.1	< 0.001	3
COI (%)	11.7	26.0	< 0.001	1,2,3
Elective (%)	7.7	2.7	< 0.001	1,2,3
Trauma (%)	36.6	23.2	< 0.001	
Surgery (%)	30.2	44.8	0.001	

Development set.

HR: Heart rate; MAP: Mean arterial pressure; (A-a)O2 gradient: Alveolar-arterial oxygen gradient; MV: Mechanical ventilation; COI: Chronic organ insufficiency; MORT: Hospital mortality; Data presented as the mean (SD) or percentages.

SCORE: (1) APACHE II, (2) SAPS II and (3) MPM II 24.

p: *D*etermined by χ^2 ^test for percentages or *Mann-Whitney *test for comparison of medians.

Only the COI variable reflected the chronic illnesses of the patient. For variables related to diagnoses, the surgery group was associated with a greater possibility of hospital mortality, while the trauma group was associated with a lower likelihood of mortality.

### Multiple Logistic Regression Model

Table 5 shows the LR model including 9 variables (Continuous: Age, HR, Glasgow and (A-a)O2 gradient. Discrete: Inotropic therapy, MV, Acute renal failure, COI and Trauma) selected from the 24 variables.

**Table 5:** Results of multiple logistic regression

Variable	Coefficient	SD	*p-value*	OR	95% CI
Age (years)	0.041	0.004	< 0.001	1.041	1.033 - 1.050
HR (ppm)	0.009	0.002	< 0.001	1.009	1.005 - 1.013
Inotropic therapy	0.730	0.137	< 0.001	2.074	1.585 - 2.714
Glasgow	-0.180	0.019	< 0.001	0.835	0.805 - 0.867
MV	0.502	0.145	0.001	1.655	1.245 - 2.201
(A-a)O2 gradient	0.002	0.001	< 0.001	1.002	1.002 - 1.003
Acute renal failure	0.459	0.160	0.002	1.582	1.180 - 2.123
COI	1.026	0.156	< 0.001	2.789	2.054 - 3.788
Trauma	-0.357	0.160	0.026	0.700	0.511 - 0.957
Intercept	-3.351				

HR: Heart rate; MV: Mechanical ventilation; (A-a)O2 gradient: Alveolar-arterial oxygen gradient; COI: Chronic organ insufficiency.

### Classification Tree Models

The variables common to the three CTs and the LR model are inotropic therapy (INOT), Glasgow value, (A-a)O2 gradient ((A-a)O2), age and COI.

**Figure 1:**
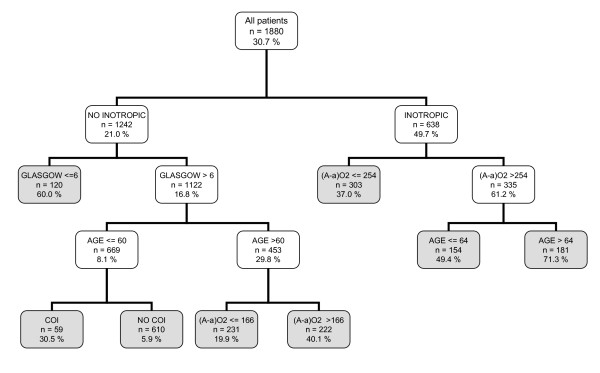
**Classification tree by CART algorithm**. The gray squares denote terminal prognostic subgroups. INOT: Inotropic therapy; (A-a)O2 gradient: Alveolar-arterial oxygen gradient (mmHg); MV: Mechanical ventilation; COI: Chronic organ insufficiency.

Figure 1 shows the CT based on the CART methodology (the three programs gave the same result). It used only five variables and began with INOT. It generated eight decision rules with an assignment rank of probability ranging from 5.9% to a maximum of 71.3%.

It is noted that a CT can use the same variables in various decision rules and that, for continuous variables, different cut-off points can be selected.

**Figure 2:**
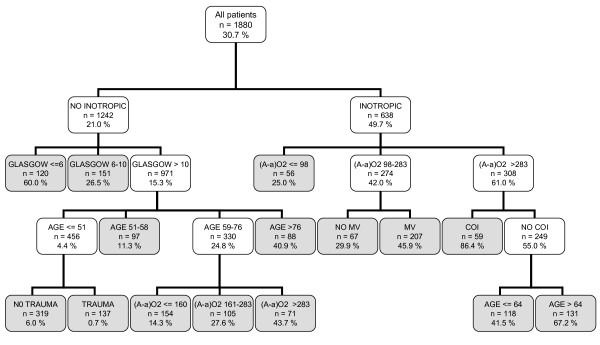
**Classification tree by CHAID algorithm**. The gray squares denote terminal prognostic subgroups. INOT: Inotropic therapy; (A-a)O2 gradient: Alveolar-arterial oxygen gradient (mmHg); MV: Mechanical ventilation; COI: Chronic organ insufficiency.

Figure 2 illustrates the CT based on the CHAID methodology. It used seven variables, and it also began with the variable INOT. It generated fifteen decision rules with an assignment rank of probability ranging from 0.7% to a maximum of 86.4%. In this type of CT, the Glasgow value, age and (A-a)O2 variables were divided into intervals with more than two possibilities.

**Figure 3:**
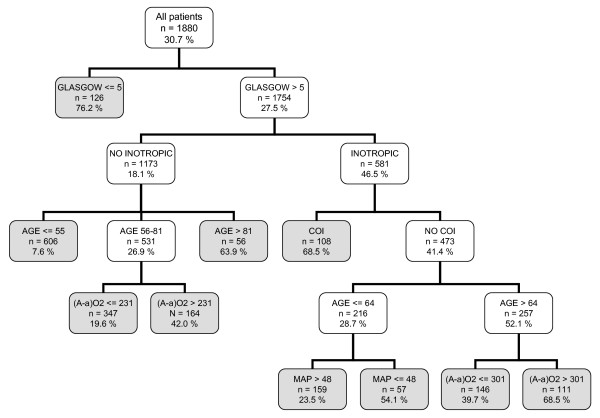
**Classification tree by C4.5 algorithm**. The gray squares denote terminal prognostic subgroups. INOT: Inotropic therapy; (A-a)O2 gradient: Alveolar-arterial oxygen gradient (mmHg); MV: Mechanical ventilation; COI: Chronic organ insufficiency; MAP: Mean arterial pressure.

Figure 3 depicts the C4.5 model, which used six variables (the five common variables and the MAP, which is not included in the LR model) and generated ten decision rules. The probabilities ranged between 7.6% and 76.2%. In contrast to the other CTs, in this CT, the first variable was the point value on the Glasgow scale.

### Comparison of model properties

The three CT models and the LR model were also compared with those generated using the APACHE II, SAPS II and MPM II-24 scores.

The severity scores were applied without making recalibration in all the population (development and validation sets).

Table 6 shows the values for the properties evaluated. It can be seen that all models achieved an acceptable discrimination (an AUC greater than 0.70) both in the development and the validation set.

**Table 6:** Performance of the classification models: development and validation sets

DEVELOPMENT (n = 1880)						
**Models**	**AUC (CI 95%)**	**HL-C**	**Brier**	**PPV (CI 95%)**	**PCC (CI 95%)**	**SMR (CI 95%)**
**APACHE II**	0.81 (0.79 - 0.83)	68.2	0.17	0.72 (0.66 - 0.78)	0.75 (0.73 - 0.77)	1.30 (1.23 - 1.37)
**SAPS II**	0.82 (0.80 - 0.84)	77.2	0.16	0.74 (0.68 - 0.79)	0.74 (0.68 - 0.75)	1.31 (1.24 - 1.38)
**MPM II 24**	0.81 (0.79 - 0.83)	74.2	0.16	0.75 (0.70 - 0.80)	0.77 (0.75 - 0.79)	1.29 (1.22 - 1.36)
**Logistic R**	0.83 (0.81 - 0.85)	16.8	0.16	0.75 (0.70 - 0.80)	0.77 (0.76 - 0.79)	1.00 (0.92 - 1.10)
**CART**	0.78 (0.76 - 0.80)	------	0.17	0.67 (0.61 - 0.72)	0.75 (0.73 - 0.77)	1.00 (0.94 - 1.06)
**CHAID**	0.80 (0.78 - 0.82)	------	0.16	0.68 (0.63 - 0.73)	0.75 (0.73 - 0.77)	1.00 (0.93 - 1.08)
**C4.5**	0.80 (0.78 - 0.82)	------	0.16	0.69 (0.65 - 0.74)	0.78 (0.76 - 0.80)	1.00 (0.94 - 1.06)

**VALIDATION (n = 804)**						

**APACHE II**	0.77 (0.74 - 0.81)	74.1	0.18	0.69 (0.60 - 0.70)	0.73 (0.70 - 0.76)	1.36 (1.26 - 1.47)
**SAPS II**	0.79 (0.76 - 0.83)	78.3	0.18	0.71 (0.63 - 0.78)	0.74 (0.71 - 0.77)	1.39 (1.28 - 1.49)
**MPM II 24**	0.79 (0.75 - 0.82)	66.9	0.18	0.71 (0.63 - 0.78)	0.74 (0.71 - 0.77)	1.36 (1.25 - 1.46)
**Logistic R**	0.81 (0.78 - 0.84)	41.5	0.17	0.73 (0.66 - 0.81)	0.75 (0.73 - 0.78)	1.22 (1.16 - 1.29)
**CART**	0.75 (0.71 - 0.81)	------	0.18	0.64 (0.57 - 0.72)	0.72 (0.69 - 0.75)	1.04 (0.95 - 1.31)
**CHAID**	0.76 (0.72 - 0.79)	------	0.18	0.64 (0.56 - 0.72)	0.72 (0.69 - 0.75)	1.06 (0.97 - 1.15)
**C4.5**	0.76 (0.73 - 0.80)	------	0.18	0.70 (0.63 - 0.76)	0.76 (0.73 - 0.79)	1.08 (0.98 - 1.16)

AUC: Area under ROC curve; CI: Confidence interval; HL-C: Hosmer-Lemeshow test C (eight degrees of freedom); Brier: Brier score; PPV: Positive predictive value (cutoff 0.5); PCC: Percentage correctly classified (cutoff 0.5); SMR: Standardized mortality ratio. The severity scores (APACHE II, SAPS II and MPM II 24) were not developed in the development phase and recalibration was not performed.

**Figure 4:**
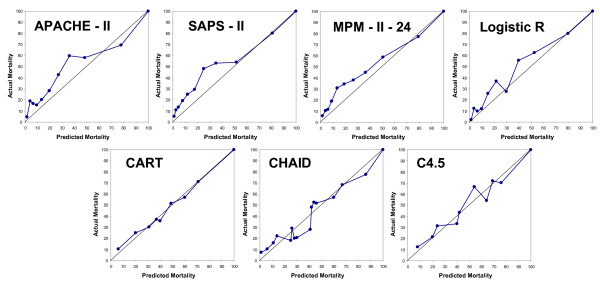
**Calibration curves for the classification models**. Validation set.

Figure 4 presents the calibration curves of the models. It is notable that some curves were displaced to the observed mortality; this coincided with an SMR greater than 1 (with a CI of 95% that does not include 1) (Table 6). The models based on the CTs were better calibrated (this was observed both in the calibration curves and in the obtained SMR (see Table 6)).

All the models correctly classified approximately 75% of the cases evaluated.

### Comparison of individual probabilities generated by the CT models

Table 7 shows the correlations between the probabilities calculated with the 3 CTs and the LR model (all of them statistically significant).

**Table 7:** Correlation matrix of the probabilities (CTs and LR models)

	DEVELOPMENT SET (n = 1880)			VALIDATION SET (n = 804)		
	**LR**	**CART**	**CHAID**	**LR**	**CART**	**CHAID**

**LR**	-------	-------	-------	-------	-------	-------
**CART**	0.872	-------	-------	0.877	-------	-------
**CHAID**	0.803	0.821	-------	0.788	0.810	-------
**C4.5**	0.768	0.796	0.789	0.777	0.794	0.801

LR: Logistic Regression. Numbers represent Spearman correlations coefficients.

All values with a p-value < 0.001.

**Figure 5:**
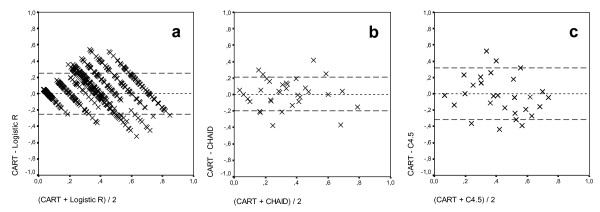
**Bland-Altman plot analysis**. (a) CART vs Logistic Regression. (b) CART vs CHAID. (c) CART vs C4.5. The dotted lines are the limits of agreement (mean ± 2 SD). Validation set.

Figure 5 shows the *Bland-Altman *results obtained in the validation set by comparing the probabilities determined by the CART CT with those of the LR, CHAID and C4.5 CTs.

We observed that there were patients for whom the difference in the probabilities exceeded the acceptable limit of the test. There were 116 patients included in the comparison of the CART and CHAID CTs, and 245 in the comparison of the CART and C4.5 CTs. The differences can be partly attributed to the behaviour of the Glasgow variable (different cut-off points or partitions) and to the influence of the COI variable in the different divisions of the tree branches.

The different models generate, in some patients, different allocation of death provability. When performing a validation with records not used in the phase of development, the different allocation of probability determines in our case a conservation of a similar discrimination but that the calibration is different (being better for the AC).

## Discussion

The results illustrate that the ICU had particular demographic characteristics due to its case mix, with a low percentage of scheduled patients, a long length of stay and high mortality. These data are important when it comes to appraising and generalising the results obtained with our database [16].

The results yielded mortality rates that were higher than expected (according to the classic APACHE II, SAPS II and MPM II-24 scores), which can be partly attributed to these individual characteristics [17]. However, this finding also necessitates a recalibration of these models in order to achieve a correct stratification of the patients' risk of hospital mortality [18].

Previously, CTs have been used with critical patients, e.g., for the calculation of the probability of death from coronary pathology [19], intracerebral haemorrhages [20] or traumatic brain injuries [21], for the prediction of persistent vegetative states [22] or (as in our study) for stratifying the probability of death in a general population of ICU patients [23,24].

The common variables selected by the three CT types (and also by the model based on LR) were: the necessity of inotropic therapy, the point value on the Glasgow coma scale, the alveolar-arterial gradient in oxygen, age and the presence of antecedents of important chronic diseases. This group of variables included information concerning chronic health and age (which were variables specific to the patient), the point value on the Glasgow coma scale and the (A-a)O2 gradient as deviations from the normal state as well as a variable specific to the intensive treatment (INOT). The selection of some of these variables has also been reported in other studies of mortality in other groups of critical patients [25].

These five variables are capable of stratifying the examined population of critical patients (for example, as in the CART CT), using eight simple decision rules, with acceptable properties of discrimination and calibration.

We also observed that the three CT types exhibited differences. Even when incorporating the five common variables mentioned earlier, these CTs differed in the first variable to be selected, in the details of "branching", in the cut-off points (and subgroups), in the order of variable selection and in the incorporation of other variables.

We have already seen that the CART CT includes the five general variables. The C4.5 CT adds the MAP (Mean Arterial Pressure) variable and the CHAID CT includes MV (Mechanical Ventilation) variables and the fact of belonging to the trauma group. The LR model uses those of the CHAID CT model, also including the Acute Renal Failure and HR (Heart Rate) variables.

The CT software allows to adjust the levels and the number of partitions for each branch in order to get more complex models [7]. In our case, our only restriction (in the 3 CT models) was that the minimum number of subjects in the terminal nodes should be of 50 patients.

We cannot state which CT was optimal (since they had similar general properties). The CART and CHAID CTs were similar in their order of partitioning, although the CHAID CT (due to its inherent characteristics) separated the continuous variables into more than two possibilities and generated more decision rules. The CART CT was simpler, while the CHAID CT showed greater complexity (and also selected more variables). Different CTs can select different first variables, and in the C4.5 CT, the first variable, the Glasgow point value, was different from that of the other CTs; the C4.5 CT also incorporated different variables. The analysis of the individual probabilities generated by the different CTs (in spite of a good correlation) assisted in the identification of possible "problem" variables, e.g. the Glasgow point value and the COI variable, in their order of appearance in the decision rules generated.

The CTs most widely used for medical applications have been based on the CART methodology, but studies that use other CT types have started to appear [26-28].

When there is a classification problem, there is no model that can be chosen *a priori *to be the best [29]. Even with the same information, different CTs develop models with different interpretations [30]. Based on our data, the CTs do not compete with the classic scores in their function of calculating individual probabilities. In the case of a large database, the CTs generated would be too complex to interpret and use with regularity (many branches and decision rules). The immediate interpretative advantage of CTs is only obtained with simple trees [31,32].

Our study had several limitations. In the first place, it was carried out in only one ICU and within a ten-year span database (although no variation was observed during the period of study). It would also have been possible to employ more methodologies for comparison or to improve those that were used, by incorporating relations and/or ranks of *a priori *variables, as do the classic scores.

As exposed by one of the reviewers, we found a great difference between the observed and expected mortality in the validation group in the LR model. The LR-based model could have been carried out using the variables as categorical, thus minimizing the possible effect that outlier values (using the variables as continuous) have on the predicted outcome. One of the advantages of CT-based models is that they automatically change the continuous variables into categorical ones and that their cut-off points could also be used to create a LR model with discreet variables

We must mention the effort at Waikato University (New Zealand) regarding the free-access program WEKA, which strives to collect (in a single tool) the majority of the methodologies that are used to classify, select and group variables [11].

There are models, with different methodologies that could improve the individual properties and achieve greater precision in classification [33,34].

The principal advantage of CTs is that they are easy to interpret. However, this advantage could turn into an obstacle, since we tend to choose the optimal CT as that which more closely approaches the clinical reasoning that coincides with that of the program user [35]. An understanding of the clinical problem is necessary in order to adequately interpret CTs.

One contribution of our effort was the demonstration that the CT methodology is not unique and that different CTs could be generated according to various methodologies. The CTs assisted in both selecting variables of greater importance in the problem of classification and determining the best cut-off points for the continual variables.

We believe that CTs (e.g., the model based on CART) are mainly useful in obtaining homogenous groups for the assignation of the probability of hospital mortality. These groups with different characteristics (defined by rules of classification that can be interpreted) can serve, for example, as a basis for the creation of new scores.

We intend to do further research including a multi-centre study, with the incorporation of more methodologies and the possible use of hybrid models. In order to generalise our results, external validation will be required [36].

## Conclusion

The main benefits to CT analysis are to identify a relatively small number of groups that are reasonably homogeneous with regard to the outcome. The CTs can be used in intensive care medicine for assisting in diagnosis and prognosis [37,38]. Those less familiar with CTs should realise that this us a class of methods including many different approaches, and that these different approaches may result in considerable differences in classifications.

## Competing interests

The authors declare that they have no competing interests.

## Authors' contributions

JT and JM provided the CT procedure, implemented the algorithms, performed statistical and drafted the manuscript. MB, LS and ARP analyzed the data, interpreted the results and wrote the final paper. All authors read and approved the final manuscript.

## Pre-publication history

The pre-publication history for this paper can be accessed here:

http://www.biomedcentral.com/1471-2288/9/83/prepub
